# Investigating the Antifibrotic Effects of β-Citronellol on a TGF-β1-Stimulated LX-2 Hepatic Stellate Cell Model

**DOI:** 10.3390/biom14070800

**Published:** 2024-07-05

**Authors:** Watunyoo Buakaew, Sucheewin Krobthong, Yodying Yingchutrakul, Pachuen Potup, Yordhathai Thongsri, Krai Daowtak, Antonio Ferrante, Kanchana Usuwanthim

**Affiliations:** 1 Department of Microbiology, Faculty of Medicine, Srinakharinwirot University, Bangkok 10110, Thailand; watunyoo@g.swu.ac.th; 2 Cellular and Molecular Immunology Research Unit (CMIRU), Faculty of Allied Health Sciences, Naresuan University, Phitsanulok 65000, Thailand; pachuenp@nu.ac.th (P.P.); yordhathait@nu.ac.th (Y.T.); kraid@nu.ac.th (K.D.); 3 Center of Excellence in Natural Products Chemistry (CENP), Department of Chemistry, Faculty of Science, Chulalongkorn University, Bangkok 10330, Thailand; sucheewin.k@chula.ac.th; 4 National Center for Genetic Engineering and Biotechnology, National Science and Technology Development Agency (NSTDA), Pathum Thani 12120, Thailand; yodying.yin@nstda.or.th; 5 Department of Immunopathology, South Australia (SA) Pathology, Women’s and Children’s Hospital, Adelaide, SA 5006, Australia; antonio.ferrante@adelaide.edu.au; 6 The Adelaide Medical School, The School of Biological Science and the Robinson Research Institute, University of Adelaide, Adelaide, SA 5000, Australia

**Keywords:** β-citronellol, *Citrus hystrix* DC., hepatic stellate cell, liver fibrosis, proteomic analysis, molecular docking

## Abstract

Liver fibrosis, a consequence of chronic liver damage or inflammation, is characterized by the excessive buildup of extracellular matrix components. This progressive condition significantly raises the risk of severe liver diseases like cirrhosis and hepatocellular carcinoma. The lack of approved therapeutics underscores the urgent need for novel anti-fibrotic drugs. Hepatic stellate cells (HSCs), key players in fibrogenesis, are promising targets for drug discovery. This study investigated the anti-fibrotic potential of *Citrus hystrix* DC. (KL) and its bioactive compound, β-citronellol (β-CIT), in a human HSC cell line (LX-2). Cells exposed to TGF-β1 to induce fibrogenesis were co-treated with crude KL extract and β-CIT. Gene expression was analyzed by real-time qRT-PCR to assess fibrosis-associated genes (*ACTA2*, *COL1A1*, *TIMP1*, *SMAD2*). The release of matrix metalloproteinase 9 (MMP-9) was measured by ELISA. Proteomic analysis and molecular docking identified potential signaling proteins and modeled protein–ligand interactions. The results showed that both crude KL extract and β-CIT suppressed HSC activation genes and MMP-9 levels. The MAPK signaling pathway emerged as a potential target of β-CIT. This study demonstrates the ability of KL extract and β-CIT to inhibit HSC activation during TGF-β1-induced fibrogenesis, suggesting a promising role of β-CIT in anti-hepatic fibrosis therapies.

## 1. Introduction

Liver disease accounts for 4% of all deaths worldwide, and the most common cause is cirrhosis [1]. Cirrhosis is an advanced stage of liver fibrosis, characterized by the irreversible formation of regenerative nodules with excessive extracellular matrix (ECM) accumulation. Liver fibrogenesis is the key mechanism in the wound-healing process of the liver after inflammation and injury, providing fibril-forming collagen as a scaffold for tissue regeneration [2]. Various intrinsic and extrinsic stimuli of hepatic inflammation, such as cholecystitis, viral hepatitis, alcohol consumption, and non-alcoholic fatty liver disease (NAFLD), lead to the activation of hepatic stellate cells (HSCs) [3]. HSCs differentiate from myofibroblasts and have been established as the central driver of liver fibrogenesis in in vitro and in vivo experiments [4]. In the homeostasis of the wound-healing process, ECM deposition is a tightly regulated process that is reversible after the stimulant is removed. Transforming growth factor-beta 1 (TGF-β1) is one of the key molecules involved in liver fibrogenesis in HSCs via the stimulation of mothers against decapentaplegic homolog (Smad) transcription factors [4]. Smad2 is pivotal transcriptional regulator within the HSCs’ response to TGF-β signaling [5]. Activated HSCs play a central role in liver fibrogenesis and ECM remodeling. This is evidenced by their expression of proteins such as alpha-smooth muscle actin (α-SMA), collagen type I alpha I, and tissue inhibitor of metalloproteinase 1 (TIMP1). Alpha-smooth muscle actin, encoded by the *ACTA2* gene, is crucial for cellular contractile function and is upregulated in myofibroblasts [6]. The *COL1A1* gene encodes collagen type I alpha I, a major component of ECM deposition in hepatic fibrosis [7]. The *TIMP1* gene encodes a protein that regulates the activity of MMPs, and its inhibition has been shown to reduce fibrotic responses [8]. The activation of HSCs during hepatic inflammation induces alterations in liver microarchitecture, disrupting hepatic cell function. This creates a microenvironment that promotes the development of various liver pathologies, including cirrhosis and cancer [9]. Patients with chronic liver fibrosis progression are at a higher risk of developing liver cancer such as hepatocellular carcinoma (HCC) [10]. At present, there is no standard treatment available for liver fibrosis [11], and novel approaches to treat this condition are a sought-after goal. Therefore, finding a novel and effective treatment could explore the possibility of a therapeutic approach.

*Citrus hystrix* DC., also known as Kaffir lime (KL), is a plant belonging to the Rutaceae family that is mainly cultivated in Asiatic countries. KL is well known as the source of unique volatile compounds as an ingredient in foods and alternative medicine. Several chemical compounds are responsible for an intense kaffir lemon odor, such as Citronellal, L-Linalool, 1,8-Cineole, and α-Terpeneol [12]. The extract and active compounds from this plant harbor several pharmacological activities, including anti-inflammatory [13,14], antioxidants [15,16], anti-microbial [17,18], and anti-cancer effects [19,20,21]. The hepatoprotective effects of KL have been reported in paracetamol-induced liver injury in mice models [22]. However, the anti-liver fibrosis activity of the KL extract and its derivative compounds remains unexplored.

In this study, we examined the anti-fibrotic effects of the crude extract and β-citronellol (β-CIT), an active compound identified from the KL leaf. β-citronellol is a monoterpenoid that has been previously reported to have anti-inflammatory, anti-fungal, and anti-cancer properties [14,20,23]. The anti-liver fibrosis activity of β-citronellol was tested in TGF-β1-induced human HSCs (LX-2 cells). Several hepatic fibrogenesis markers were measured, including genes and protein of HSCs. Proteomics and molecular docking analyses were employed to elucidate the possible β-citronellol mechanisms of action on LX-2 cells. Our study suggested the potential role of KL extract and its compound in preventing liver fibrosis development in the HSC model, providing a new source for anti-hepatic fibrosis research.

## 2. Materials and Methods

### 2.1. Preparation of Crude Extract and Active Compound

The initial crude extract of KL leaves employed in this investigation was generated via the maceration of KL powder, as detailed in a prior publication [14]. Briefly, a 1000 g sample of KL powder underwent sequential maceration in *n*-hexane (absolute), ethyl acetate (95%), and ethanol (95%) for periods of three days each. Following filtration and evaporation of the ethanolic solute, 100.56 g of crude ethanolic KL leaf extract was obtained. The active compound β-citronellol was acquired from a commercial chemical supplier. Stock solutions of both the crude extract and the active compound were formulated using a solvent mixture of dimethyl sulfoxide (DMSO) (VWR Life Science, Radnor, PA, USA) and Tween-80 (Loba Chemie, Mumbai, India) (1:1 ratio). Throughout cell treatment procedures, the residual solvent concentration was maintained below 0.5%.

### 2.2. Cell Culture

LX-2 cells (an immortalized human hepatic stellate cell line) were cultured in Dulbecco’s Modified Eagle Medium (DMEM) supplemented with 2% fetal bovine serum (FBS) and 1% antibiotic–antimycotic solution (Thermo Fisher Scientific, Waltham, MA, USA). The cells were maintained in a humidified incubator at 37 °C with 5% CO_2_. This cell line was kindly provided by Dr. Saranyapin Potikanond (Department of Pharmacology, Chiang Mai University, Thailand).

### 2.3. Cell Viability Assay

To establish the optimal treatment concentrations of crude KL and β-CIT on LX-2 cells, a resazurin reduction cytotoxicity assay was conducted. LX-2 cells were seeded (2 × 10^4^ cells/well) in a 96-well plate and treated with varying concentrations of KL and β-CIT (0–1000 µg/mL). Following 24 h incubation at 37 °C, resazurin (MilliporeSigma, Burlington, MA, USA) (25 µg/mL) was added, and fluorescence was measured after 4 h (excitation/emission: 560/590 nm) using an EnSpire^®^ Multimode microplate reader (PerkinElmer, Waltham, MA, USA). GraphPad Prism version 8 (GraphPad Software, La Jolla, CA, USA) was used to determine inhibitory concentrations (IC). The IC_10_ value was selected as the maximum safe concentration for further experimental use.

### 2.4. RNA Extraction and Gene Expression Analysis

To assess the potential anti-fibrotic effects of KL and β-CIT on gene expression, LX-2 cells (2 × 10^5^ cells/well) were seeded into 24-well plates and stimulated with TGF-β1 (10 ng/mL) to induce fibrogenesis. Following this, the cells were treated with selected concentrations of both compounds and incubated for 24 h. After the treatment period, the supernatant was discarded, and RNA extraction was performed using Trizol reagent (Thermo Fisher Scientific, Waltham, MA, USA) in adherence to the manufacturer’s instructions. RNA concentration and purity were assessed utilizing a Nanodrop spectrophotometer. Subsequently, cDNA synthesis was performed on the extracted RNA from each condition using a Tetro™ cDNA Synthesis Kit (Meridian Bioscience, Cincinnati, OH, USA). Quantitative real-time polymerase chain reaction (real-time qPCR) was employed to evaluate the expression levels of the genes implicated in liver fibrosis. Amplification of gene transcripts was achieved using nucleotide primers designed specifically for *ACTA2*, *TIMP1*, *COL1A1*, and *SMAD2* genes. The qPCR reactions were carried out with a SensiFAST™ SYBR^®^ No-ROX Kit (Meridian Bioscience, Cincinnati, OH, USA) on a CFX96 Touch Real-Time PCR Detection System (Bio-Rad Laboratories, Hercules, CA, USA). The PCR protocol consisted of an initial polymerase activation step (95 °C, 1 min), followed by 45 cycles comprising denaturation (95 °C, 10 s) and annealing/extension (60 °C, 1 min). The 2^(−∆∆Ct)^ [24] method was used to determine the relative expression of each target gene, normalized against the internal reference gene, *GAPDH*. Table 1 provides a comprehensive list and references of the forward and reverse primers used for each gene. The equation to calculate the relative expression of specific genes is shown below.

Relative gene expression = 2^−∆∆CT^

where:

∆CT = CT (a target gene) − CT (a reference gene);

∆∆CT = ΔCT (a target sample) − ΔCT (a reference sample).

**Table 1:** The PCR primer of each gene in this study.

Gene	Description	Forward Primer (3′ → 5′)	Reverse Primer (3′ → 5′)	Ref
*ACTA2*	actin alpha 2, smooth muscle	CATCCTCATCCTCCCTTGAG	ATGAAGGATGGCTGGAACAG	[25]
*COL1A1*	Collagen type I alpha 1 chain	CCGGCTCCTGCTCCTCTTAGCG	CGTTCTGTACGCAGGTGATTGGTGG	
*TIMP1*	TIMP metallopeptidase inhibitor 1	CAAGATGTATAAAGGGTTCCAAGC	TCCATCCTGCAGTTTTCCAG	
*GAPDH*	Glyceraldehyde-3-phosphate dehydrogenase	ATGACATCAAGAAGGTGGTG	CATACCAGGAAATGAGCTTG	
*SMAD2*	SMAD family member 2	TGCTCTGAAATTTGGGGACTGA	GACGACCATCAAGAGACCTGG	[26]

### 2.5. Measurement of the MMP-9 Production

The activation of hepatic stellate cells (HSCs) is characterized by the release of matrix metalloproteinase 9 (MMP-9), a pivotal enzyme in this process. To assess whether KL and β-CIT exhibit MMP-9 inhibitory activity, LX-2 cells (2 × 10^5^ cells/well) were seeded in 24-well plates and stimulated with TGF-β1 (10 ng/mL), either alone or in conjunction with KL and β-CIT, for 24 h. Quantification of MMP-9 in the collected supernatants was performed using a human MMP-9 ELISA kit, adhering to the manufacturer’s protocol. Absorbance readings were analyzed using GraphPad Prism version 8 to determine MMP-9 levels.

### 2.6. LC-MS/MS Analysis

#### 2.6.1. Preparation of Protein Sample

To rigorously assess the impact of KL and β-CIT on TGF-β1-induced LX-2 cell global protein expression, liquid chromatography with tandem mass spectrometry (LC-MS/MS) was employed. Cellular treatment and lysate extraction procedures were consistent with those outlined in the previous section. LC-MS/MS sample preparation followed an established protocol with minor adjustments to ensure analytical reproducibility [27]. Protein concentration was attained through the use of a 3 kDa molecular weight cut-off filter. Subsequent precipitation was induced with ice-cold acetone (1:5 *v*/*v*). The resulting pellet was then resuspended in a solution of 0.3% RapidGest SF and 2.5 mM ammonium bicarbonate. A 30 µg protein aliquot was subjected to tryptic digestion. To facilitate proteolysis, disulfide bonds were reduced with 1 mM tris(2-carboxyethyl) phosphine (TCEP) at 37 °C for 2 h, followed by alkylation with 5 mM iodoacetamide (IAA) at room temperature for 50 min in a light-protected environment. The sample underwent desalting usingZeba Spin Column (Thermo Fisher Scientific, Waltham, MA, USA) before a second tryptic digestion step (1:40 enzyme-protein ratio) at 37 °C for 6 h. Finally, the digested peptides were dried and resuspended in 0.1% formic acid in preparation for LC-MS/MS analysis.

#### 2.6.2. Proteomic Data Analysis

Protein samples underwent analytical scrutiny via a high-fidelity hybrid Quadrupole-Orbitrap system (HF-X) coupled with an EASY-nLC1000 for refined separation. A nano C18 column operating in positive ionization mode facilitated chromatographic resolution. Employing a calibrated gradient of 90% acetonitrile/0.1% formic acid over 135 min (300 nL/min), with mobile phase A consisting of 0.1% formic acid in water, optimal separation was achieved. The column was meticulously regenerated and re-equilibrated. Data-dependent acquisition (TopN15) utilizing higher-energy collisional dissociation (29 eV) directed peptide analysis. Proteome Discoverer™ 2.4 software integrated MS parameters and thorough database searches (UniProt Homo sapiens, 14 January 2023). Stringent peptide/protein tolerances, modifications, and a 1% FDR ensured data integrity. After normalization (total intensity count), pathway analysis was carried out employing PADOG within Reactome v84 (Homo sapiens, 25 February 2023) [28].

### 2.7. Molecular Docking Analysis

Computational molecular docking analysis was performed to investigate the interaction between β-CIT and selected proteins implicated in HSCs’ activation, a key process in liver fibrosis. Protein 3D structures associated with HSC activation during hepatic fibrosis were obtained from the RCSB Protein Data Bank (RCSB PDB) [29,30] and prepared in PDB format. The chemical structure of β-citronellol (PubChem CID 8842) was retrieved from PubChem [31]. The protein–ligand blind docking analysis was conducted using the CB-Dock2 server https://cadd.labshare.cn/cb-dock2/index.php (accessed on 27 February 2023) [32]. Prior to docking analysis, both the protein targets and β-citronellol were prepared using the DockPrep tool within UCSF Chimera alpha software version 1.18 [33]. Post-docking, interactions between the target proteins and ligand were visualized in 2D using BIOVIA Discovery Studio Visualizer version 21.1.0.20298 (Waltham, MA, USA).

### 2.8. Statistical Analysis

Each in vitro experimental procedure was conducted in triplicate. Unless otherwise specified, data representation follows a mean ± SD format. Statistical significance between means was assessed using one-way ANOVA (Tukey’s multiple comparison test) within GraphPad Prism software (version 8.0.1). A *p*-value threshold of <0.05 denoted statistical significance.

## 3. Results

### 3.1. Cytotoxicity of Crude Extract and Active Compound on the LX-2 Cell Line

**Figure 1:**
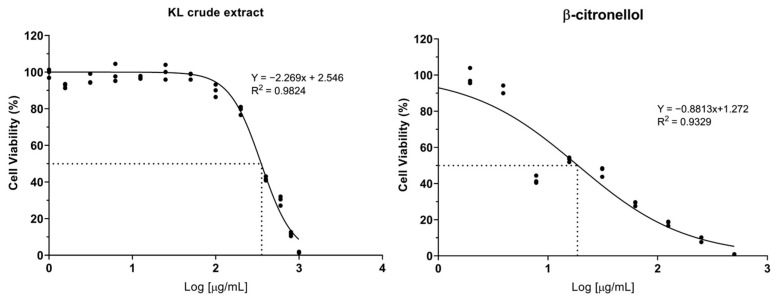
Dose–response curve of crude KL extract and β-citronellol on viability of the LX-2 cells.

The cytotoxic potential of crude KL extract and its constituent compound, β-CIT, was assessed on the LX-2 cell line through the resazurin reduction assay. Dose–response curves were generated to determine IC_5_, IC_10_, and IC_50_ values. The results demonstrated that the crude extract exhibited lower cytotoxicity relative to β-CIT, yielding IC_50_ values of 351.80 µg/mL and 18.72 µg/mL, respectively (Figure 1). Furthermore, crude KL extract displayed IC_5_ and IC_10_ values of 96.03 µg/mL and 133.48 µg/mL, while β-CIT exhibited notably lower values of 0.66 µg/mL and 1.54 µg/mL. Based on these findings, subsequent experiments investigating effects on LX-2 cells were conducted using maximum concentrations of 120 µg/mL for the crude extract and 1 µg/mL for β-CIT.

### 3.2. The Inhibition of Activated HSCs’ Genes’ Expression and MMP-9 Production

**Figure 2:**
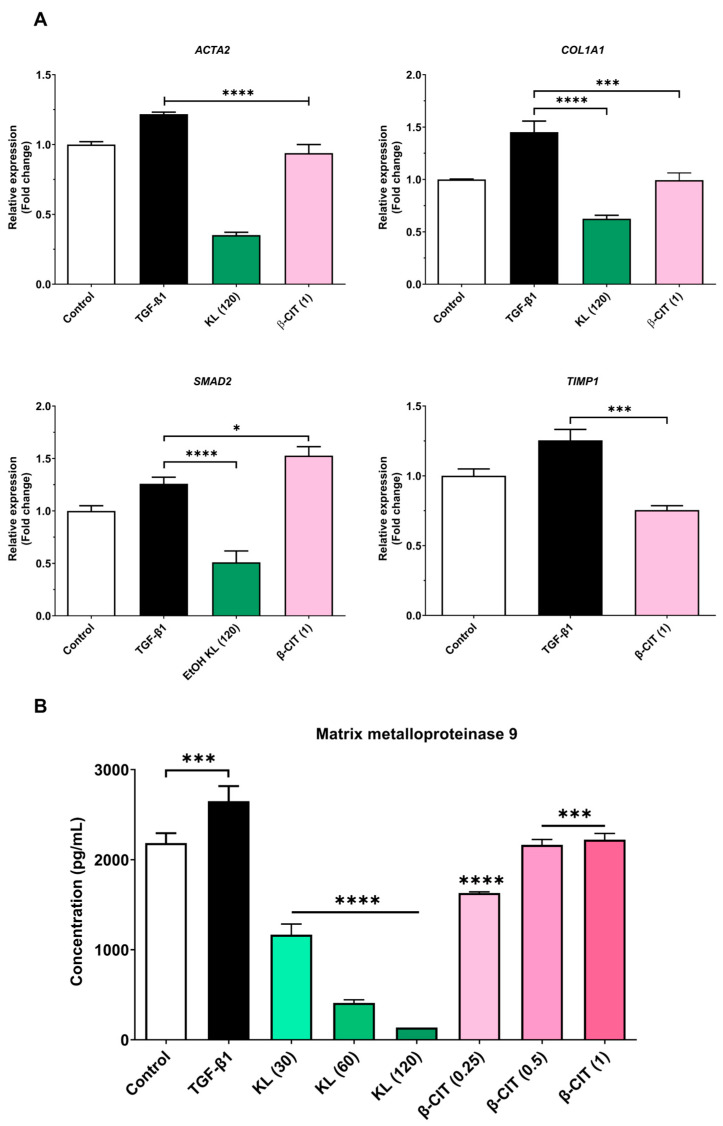
Crude KL extract and β-CIT attenuate the expression of hepatic-fibrosis-associated genes and mitigate MMP-9 production in LX-2 cells challenged with TGF-β1. Following a 24 h co-treatment protocol, mRNA was extracted and subjected to real-time qRT-PCR analysis. Expression levels were normalized to the housekeeping gene *GAPDH* (**A**), revealing a marked downregulation of fibrosis-related genes. Furthermore, a statistically significant reduction in MMP-9 levels was observed in the cell culture supernatant (**B**). Statistical significance was denoted as follows: *p* < 0.0332 (*), *p* < 0.0002 (***), *p* < 0.0001 (****).

Quantitative real-time polymerase chain reaction (qRT-PCR) elucidated the downregulation of key hepatic fibrosis markers (*ACTA2*, *COL1A1*, and *SMAD2*) upon exposure to crude KL extract. Similarly, β-CIT treatment demonstrated the inhibition of *ACTA2*, *COL1A1*, and *TIMP1* gene expression. Analysis of MMP-9 levels via the ELISA kit revealed a marked, dose-dependent reduction following exposure to crude KL extract, with a comparable decrease observed with β-CIT treatment. These findings collectively point towards antifibrotic potential in both compounds, as evidenced by their capacity to attenuate TGF-β1-induced fibrogenesis in the LX-2 cell model (Figure 2).

### 3.3. Proteomic Analysis of the Effect of β-Citronellol on LX-2 Cell

**Figure 3:**
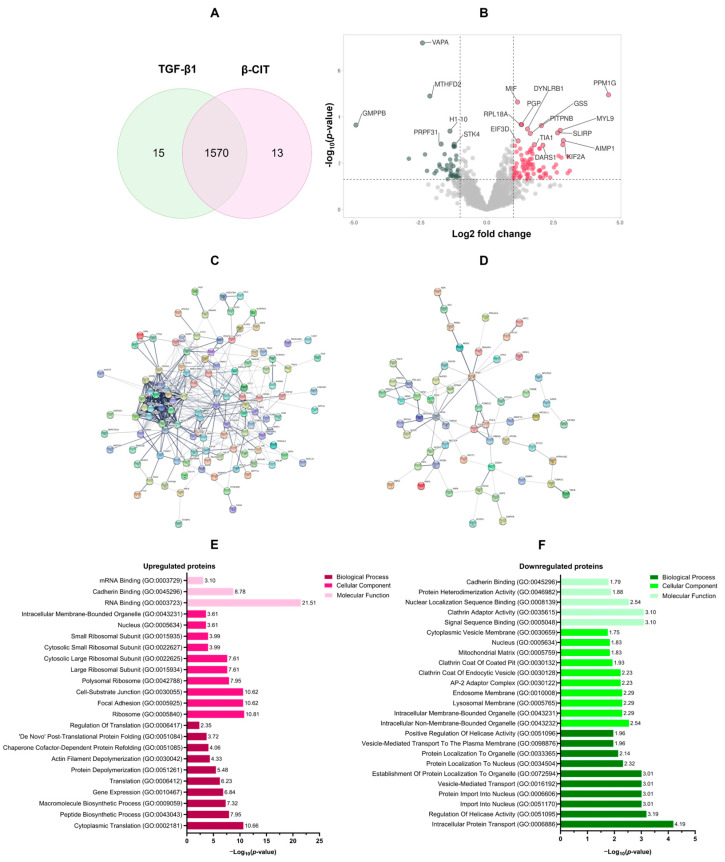
The functional enrichment analysis of DEPs. Quantitative LC-MS/MS analysis yielded a total of 1570 differentially expressed proteins (DEPs) (**A**). Volcano plots visually demonstrated the upregulated (n = 125, red) and downregulated (n = 65, green) proteins within this dataset (**B**). To elucidate potential functional relationships, protein–protein interaction (PPI) network analyses were performed separately for the upregulated (**C**) and downregulated (**D**) protein subsets. Gene ontology (GO) annotations illuminated key biological processes associated with both upregulated (**E**) and downregulated (**F**) DEPs.

To elucidate the impact of β-CIT on TGF-β1-induced LX-2 cells, a 48 h treatment regimen with β-CIT was implemented. Subsequent protein extraction and LC-MS/MS analysis yielded a total of 1570 overlapping proteins present in both TGF-β1 treatment alone and β-CIT co-treatment conditions (Figure 3A). Rigorous filtering parameters were applied (log2 fold change of ±1.5 and *p*-value < 0.05), resulting in the identification of 125 upregulated and 65 downregulated proteins (Figure 3B). A list of differentially expressed proteins (DEPs) is shown in Table 2. Further analysis of these DEPs was conducted utilizing protein–protein interaction (PPI) network visualization, revealing intricate relationships among the DEPs (Figure 3C,D).

Gene ontology (GO) annotation of differentially expressed proteins (DEPs) revealed distinct functional profiles for those exhibiting upregulation versus downregulation. Biological process enrichment demonstrated a clear emphasis on protein synthesis within the upregulated group, including cytoplasmic translation (GO:0002181), peptide biosynthesis (GO:0043043), macromolecule biosynthesis (GO:0009059), gene expression (GO:0010467), and overall translation (GO:0006412). Conversely, downregulated proteins displayed association with processes governing intracellular transport (GO:0006886), helicase regulation (GO:0051095), nuclear import (GO:0051170 and GO:0006606), and vesicle-based transport (GO:0016192). Cellular component analysis further elucidated this contrast. Upregulated proteins were overrepresented in ribosomal structures (GO:0005840, GO:0042788, GO:0015934), alongside focal adhesions (GO:0005925) and cell-substrate junctions (GO:0030055). Downregulated proteins were preferentially associated with both membrane-bound (GO:0043231) and non-membrane-bound (GO:0043232) intracellular organelles, as well as components of the lysosomal (GO:0005765), endosomal (GO:0010008), and AP-2 adaptor complex (GO:0030122). Molecular function categories mirrored these distinctions. Upregulated proteins exhibited enrichment for various RNA binding functions (GO:0003723, GO:0003729), cadherin binding (GO:0045296), signal sequence binding (GO:0005048), and activities related to clathrin adaptor function (GO:0035615). Downregulation encompassed proteins involved in nuclear localization sequence binding (GO:0008139), protein heterodimerization (GO:0046982), and cadherin binding (GO:0045296). These findings are summarized in Figure 3E,F.

**Figure 4:**
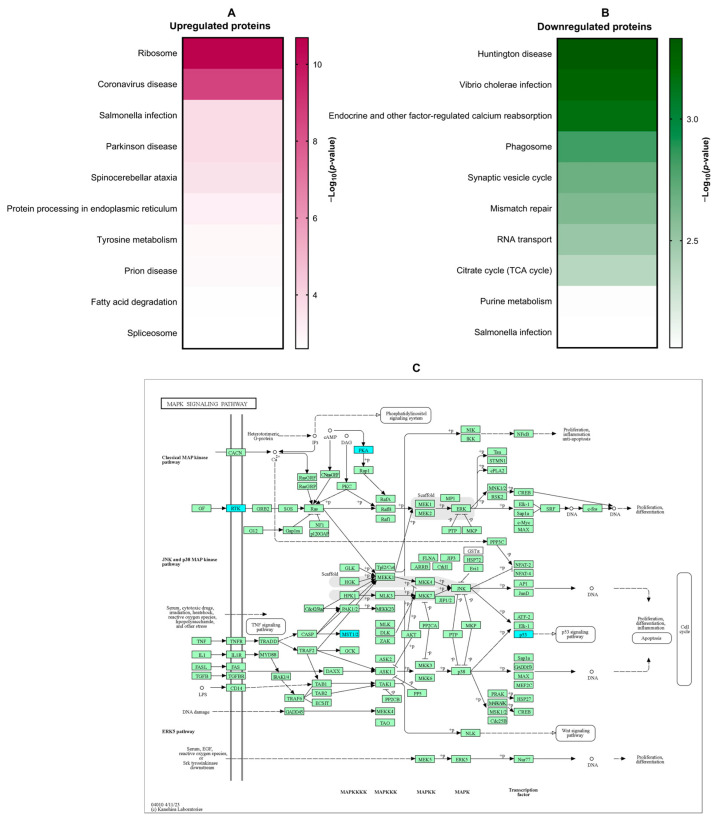
The KEGG analysis of DEPs. Amongst the most significantly enriched KEGG signaling pathways, differential protein expression patterns emerged (upregulated: **A**; downregulated: **B**). Notably, the MAPK signaling pathway exhibited a pronounced association with downregulated proteins (**C**; indicated in blue).

KEGG pathway enrichment analysis was conducted to identify signaling cascades potentially modulated by β-citronellol treatment in LX-2 cells. Enriched pathways included those associated with ribosome function, coronavirus disease, Salmonella infection, Parkinson’s disease, and spinocerebellar ataxia (Figure 4A). Downregulated proteins showed significant involvement in Huntington’s disease, Vibrio cholerae infection, endocrine-regulated calcium reabsorption, phagosome formation, and synaptic vesicle cycling (Figure 4B). The MAPK signaling pathway (Figure 4C), a known contributor to hepatic fibrosis, was a focus of further examination, with downregulated proteins highlighted in blue.

### 3.4. In Silico Molecular Docking Analysis

**Figure 5:**
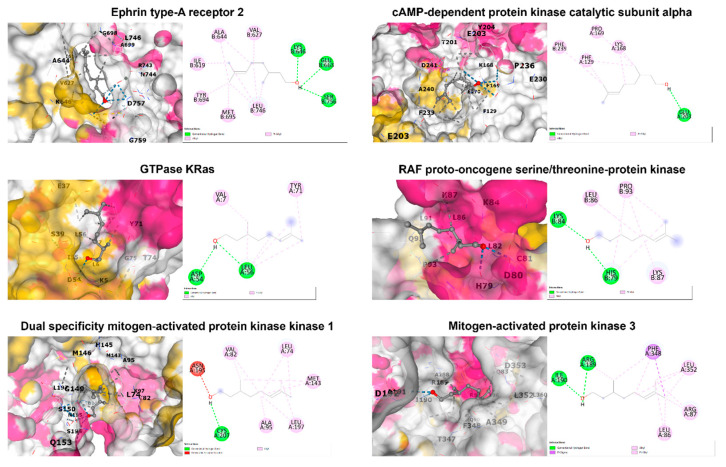
The 2D and 3D structures of candidate target proteins and β-CIT interaction.

Binding interactions between β-citronellol and potential protein targets were examined using the cavity detection-guided blind docking capabilities of the CB-Dock2 web-based platform. Detailed results, including Autodock Vina binding scores and the amino acids involved in the interactions, are outlined in Table 3. A visualization of the protein–ligand interactions in 2D and 3D formats can be found in Figure 5. Of particular interest, β-citronellol displayed favorable binding scores (−4 to −6 Kcal/mol) with target proteins known to participate in the MAPK signaling pathway.

## 4. Discussion

Considering the antecedent stage of irreversible cirrhosis, liver fibrosis arises from sustained liver injury and predisposes individuals to grave complications such as portal hypertension, hepatic encephalopathy, and hepatocellular carcinoma. The asymptomatic presentation of hepatic fibrosis, coupled with diagnostic challenges, frequently allows for unchecked progression, even though the condition may be reversible upon removal of causative factors. Furthermore, the lack of a singular, targeted medication for liver fibrosis highlights the critical need for continued research in the development of effective anti-fibrotic agents.

Hepatic stellate cell (HSC) activation is a pivotal factor in the pathogenesis of liver fibrosis. Transforming growth factor beta-1 (TGF-β1) serves as a highly effective activator of hepatic stellate cells (HSCs) via dual signaling mechanisms: the canonical (Smad-dependent) pathway and the non-canonical (non-Smad) pathway. The TGF-β1/Smad signaling cascade represents a well-documented process central to HSC activation during liver damage. Upon binding to its specific receptor, TGF-β type II receptor (TβRII), TGF-β1 initiates the recruitment of the TGF-β type I receptor (TβRI). TβRII subsequently activates TβRI through phosphorylation of serine residue, facilitating the phosphorylation of substrates like Smad2 and Smad3. Phosphorylated Smad2 and Smad3 then complex with Smad4, translocating into the nucleus. This Smad2/3/4 complex directly interacts with DNA, modulating the expression of key genes such as *ACTA2*, *COL1A1*, and *TIMP1* [34]. Our findings demonstrate that treatment of HSCs with crude KL extract and β-CIT effectively attenuates the transcriptional expression of these markers, implying a direct interference with the canonical pathway of TGF-β1-driven signaling cascade.

Proteomic analysis elucidated proteins and signaling pathways potentially mediating the inhibitory effect of β-CIT on TGF-β1-induced HSCs. The KEGG database identified several signaling pathways, especially the downregulated pathways, including ribosome function, coronavirus disease, Salmonella infection, Parkinson’s disease, and spinocerebellar ataxia. The mitogen-activated protein kinase (MAPK) signaling pathway is one of the non-canonical pathways in the HSC activation by the TGF-β1 [35]. The MAPK pathway is known to regulate diverse cellular functions such as proliferation, differentiation, survival, apoptosis, and inflammation. This pathway comprises three signaling cascades: ERK, JNK, and p38. Extensive research implicates this pathway in hepatic fibrosis through its influence on HSC behavior. MAPK signaling modulates proliferation, migration, and ECM synthesis/deposition following stimulation by growth factors like TGF-β1 via tyrosine kinases (RTKs) [36]. Our proteomic analysis demonstrated downregulation of the RTK proteins, Eph receptor A2 (*EPHA2*), alongside the MAPK signaling proteins kinase cAMP-activated catalytic subunit alpha (*PRKACA*). Eph receptors are the largest known receptor tyrosine kinases in mammals, regulating numerous biological activities of the cells, including cell adhesion, migration, proliferation, and immune cell activation [37]. The expression of Eph receptors has been linked to the progression of liver fibrosis [38]. *PRKACA* is a member of the PKA family, and has a role to transfer phosphate groups to serine or threonine residues on substrate proteins, which can alter their function and activity. It is involved in various cellular processes, including metabolism, gene expression, and cell proliferation. Some studies revealed that *PRKACA* exhibits hyperkinase activity in fibrotic liver samples from both humans and mice. This suggests that *PRKACA* may play a significant role in the development and progression of hepatic fibrosis [39]. Molecular binding analysis indicated favorable affinities between these proteins and β-CIT. These findings suggest that the anti-hepatic fibrosis potential of β-CIT may involve targeting the MAPK signaling pathway, though further studies are necessary to confirm the precise mechanism. While the TGF-β1-stimulated HSC cell line is a useful and widely accepted model to explore the mechanisms of fibrosis, screen for therapeutic agents, and understand the cellular responses to fibrogenic stimuli, our findings need to be complemented with in vivo studies to fully understand the effects of the compounds in the context of the complexities of liver fibrosis.

## 5. Conclusions

This study presents the first evidence of the potential anti-hepatic fibrotic activity of β-CIT. We demonstrate that β-CIT inhibits hepatic stellate cell activation induced by the TGF-β1 model. Mechanistically, β-CIT appears to target the canonical TGF-β1/Smad signaling pathway, along with reducing MMP-9 production—all major contributors to extracellular matrix (ECM) deposition. Further proteomic analysis and molecular docking suggest a potential additional mode of action involving MAPK tyrosine kinase signaling proteins. While the TGF-β1-stimulated HSC cell line is a useful and widely accepted model to explore the mechanisms of fibrosis, screen for therapeutic agents, and understand the cellular responses to fibrogenic stimuli, our findings need to be complemented with in vivo studies to fully understand the effects of this compound in the context of the complexities of liver fibrosis.

## Figures and Tables

**Table 2:** The top 10 expression proteins in both upregulated and downregulated manners.

Uniprot ID	Gene ID	Description	Log2 Fold Change	−Log10 *p*-Value
Top 10 upregulated proteins				
O15355	*PPM1G*	Protein phosphatase, Mg^2+^/Mn^2+^ dependent 1G	4.57	4.95
Q9Y4W6	*AFG3L2*	AFG3-like protein 2	3.11	1.68
P04066	*FUCA1*	Tissue alpha-L-fucosidase	3.05	2.29
Q16537	*PPP2R5E*	Serine/threonine-protein phosphatase 2A 56 kDa regulatory subunit epsilon isoform	3.04	1.57
Q12904	*AIMP1*	Aminoacyl tRNA synthase complex-interacting multifunctional protein 1	2.87	2.98
O00139	*KIF2A*	Kinesin-like protein KIF2A	2.85	2.80
Q16186	*ADRM1*	Proteasomal ubiquitin receptor ADRM1	2.80	2.24
P24844	*MYL9*	Myosin regulatory light polypeptide 9	2.76	3.42
Q0VDF9	*HSPA14*	Heat shock 70 kDa protein 14	2.69	2.32
Q9GZT3	*SLIRP*	SRA stem-loop-interacting RNA-binding protein, mitochondrial	2.66	3.31
Top 10 downregulated proteins				
Q9Y5P6	*GMPPB*	Mannose-1-phosphate guanyltransferase beta	−4.91	3.65
O75431	*MTX2*	Metaxin-2	−2.92	2.20
Q9P0L0	*VAPA*	Vesicle-associated membrane protein-associated protein A	−2.42	7.19
P04264	*KRT1*	Keratin, type II cytoskeletal 1	−2.32	2.38
O76021	*RSL1D1*	Ribosomal L1 domain-containing protein 1	−2.24	1.67
P13995	*MTHFD2*	Bifunctional methylenetetrahydrofolate dehydrogenase/cyclohydrolase, mitochondrial	−2.14	4.90
P51398	*DAP3*	28S ribosomal protein S29, mitochondrial	−1.96	1.56
Q9P2R3	*ANKFY1*	Rabankyrin-5	−1.89	1.66
O00291	*HIP1*	Huntingtin-interacting protein 1	−1.78	1.95

**Table 3:** The Autodock Vina binding score and interaction amino acid of selected proteins.

PDB ID	Protein Name	Binding Score (Kcal/mol)	Interacting Amino Acid
1MQB	Ephrin type-A receptor 2	−5.0	Ile619, Val627, Ala644, Lys646, Glu663, Tyr694, Met695, Leu746, Ser756
7Y1G	cAMP-dependent protein kinase catalytic subunit alpha	−5.4	Phe129, Lys168, Pro169, Glu203, Phe239
8FMI	GTPase KRas	−4.8	Val7, Asp54, Leu56, Tyr71
1C1Y	RAF proto-oncogene serine/threonine-protein kinase	−4.2	His79, Lys84, Leu86, Lys87, Pro93
1S9J	Dual specificity mitogen-activated protein kinase kinase 1	−5.3	Leu74, Val82, Ala95, Met143, Asn195, Leu197, Cys207
2ZOQ	Mitogen-activated protein kinase 3	−5.7	Leu86, Arg87, Arg189, Ile190, Phe348, Leu352

## Data Availability

The original data presented in the study are openly available in ProteomeXchange Consortium via the PRIDE partner repository at http://www.ebi.ac.uk/pride/archive/PXD052806 (accessed on 6 April 2024).
